# Medical History from the Earliest Times

**Published:** 1892-07-02

**Authors:** E. T. Withington


### Medical History from the Earliest Times.

By E. T. Withington, M.A., M.B. Oxon.

### XV.—MILITARY MEDICINE IN ANCIENT GREECE.

Dr. H. Fröhlich, whose interesting essays on Homeric Medicine have already been noticed, has come to the conclusion that the poet himself was nothing less than an army surgeon. This will probably add another to the many disputed Homeric questions, but we will not venture to discuss it here; nor need we repeat what has already been said about the surgeons who followed Agamemnon.

In Greece itself we naturally look for our earliest information to the great military state, Lacedæmon, and among the laws ascribed to Lycurgus was one which provided that army surgeons should retire to a safe place during a battle. Xenophon also says that the medical men who accompanied the Spartan armies shared the same tents with the "peers," the flute players, the priests, and any volunteers who might be present, which seems highly respectable company. We have, however, no information as to who these surgeons were, or what the nature of their qualifications, and indeed know curiously little about the physicians of Greece proper as distinct from her colonies. But not impossibly some of the youth of Sparta may have found the law of Lycurgus respecting surgeons more attractive than that ordinance which forbade the men of Laconia to retreat when in face of the enemy.

One of the oldest authentic records of Greek military medicine dates from the period of the Persian wars, about B.C. 450. This is an inscription found at Dali in Cyprus, the translation of which has much exercised philologists. We learn from it that when the men of Idalion went forth to repel an inroad of Persians and Kitians, a certain physician, Onasilos, and his pupils (or brothers?) went with them and tended the wounded free of charge; wherefore the Demos decrees that rewards shall be given (1) to Onasilos and his pupils, (2) to Onasilos himself separately. In each case money and lands are mentioned, but for some reason they are not to have both, and the amounts given cannot be translated. The rest of the inscription is very imperfect, but from its conclusion we gather that Onasilos received a landed estate, free from taxes, for himself and his heirs.

Such volunteering appears to have been frequent in later times, for a Hippocratic writer advises the young surgeon to seek opportunities of following a military expedition, and remarks that there are works specially devoted to army surgery. It may have been on such an occasion that Diocles invented his graphiscus, a spoon-like instrument for extracting embedded weapons, afterwards much used in the Roman army.

An ancient though spurious document declares that Hippocrates sent his son Thessalus with the expedition to Sicily. "When you were consulting about a surgeon to accompany the expedition," says the imaginary Thessalus, who is supposed to be addressing the Athenians, "my father offered to send me, to fit me out at his own expense, and to ask no pay till the expedition started." May we venture to conclude from this that at the time of the writer there was a paid army medical service, and that this payment was, in the case of pupils, given to their master? The mention of one surgeon for the whole expedition, however, makes us suspect that the account is entirely imaginary.

The immortal Ten Thousand were accompanied by at least eight surgeons, for Xenophon mentions this number as having been left behind on one occasion during the retreat to look after the wounded.

In the Macedonian age historians, unfortunately, give us only anecdotes, as, for instance, of the surgeon who extracted an arrow from Philip's eye, or of that other practitioner who received the flattering token of confidence from Alexander the Great. It is indeed pleasant to learn that the king so trusted his physician as to drink the supposed poison, while holding in his hand the accusation against him, but we should have been glad to have also heard something of the arrangements for the wounded of that army which conquered Western Asia, and which was accompanied by the most celebrated practitioners from all parts of Greece. Besides Alexander's own physician, Philip of Acarnania, we hear of Callisthenes of Olynthus, whose outspoken criticism of the king's claims to adoration cost him his life, Critodemus the Asclepiad of Cos, Glaucias, who was crucified for failing to cure Hephæstion, Pausanias, Alexippus, and others. Callisthenes wrote a history of the expedition, which may have contained interesting medical details, but only a few fragments have survived.

Finally, when Sparta fought her last battle for the freedom of Hellas, and saw her warriors driven in no ignoble defeat from the hill of Sellasia, we are told that every house opened its doors, and that all who were left in Lacedæmon united in refreshing the soldiers and binding up their wounds. Thus the story of the military medicine of independent Greece ends with the city whence it began, but we shall soon find Greek surgeons actively employed in Roman armies, and on reaching that wider development of Hellenic civilisation known as the Byzantine Empire, shall come upon an army medical service such as Lycurgus never dreamt of, and not unworthy to be compared even with that of modern times.

